# Effect Of Exercise on Muscle Mass, Fat Mass, Bone Mass, Muscular Strength and Physical Performance in Community Dwelling Older Adults: Systematic Review and Meta-Analysis

**DOI:** 10.14336/AD.2022.0215

**Published:** 2022-10-01

**Authors:** Alejandra González-Rocha, Lucia Mendez-Sanchez, María Araceli Ortíz-Rodríguez, Edgar Denova-Gutiérrez

**Affiliations:** ^1^Universidad Iberoamericana, Ciudad de México, México.; ^2^Centro de Investigación en Nutrición y Salud, Instituto Nacional de Salud Pública, Cuernavaca, México.; ^3^Unidad de Investigación en Epidemiología Clínica, Hospital Infantil de México Federico Gómez-UNAM, Ciudad de México, México.; ^4^Facultad de Nutrición, Universidad Autonoma del Estado de Morelos, Cuernavaca, México.

**Keywords:** muscle mass, fat mass, physical performance, muscular strength, older adults, Meta-analysis

## Abstract

The demographic transition makes it necessary to establish new recommendations about the components that are most affected by aging, such as: muscle mass, fat mass, bone mass, muscle strength, and physical performance. Exercise has been identified as a factor that improves those conditions. The aim of this review is to synthetize and analyze the results of exercise interventions on muscle mass, fat mass, bone mass, strength and physical performance in community dwelling older adults (OA). A systematic search was carried out in eleven databases, using validated terms as “aged”, “exercise” and others. For the meta-analysis, we differentiated by type of exercise and outcome. Twenty-nine randomized clinical trials were obtained for the review and 24 clinical trials for meta-analysis. This study identified an increase of 1.0 kg (95% Confidence Interval [CI] 0.3 -1.7) in total muscle mass (TMM) and 0.4 kg (95%CI 0.0,0.7) in appendicular muscle mass (AMM); a decrease of -3.7 kg (95% CI -5.8, -1.5) in total fat mass and -3.7% (95%CI -5.8, -1.5) in fat percentage after the resistance exercise intervention by 2-3 times per week. A -3.0% (95%CI -4.6, -1.3) decrease was observed in fat percentage after the aerobic exercise intervention. The quality of the evidence was ranked from high to very low; the risk of bias most common was performance bias and other bias. This study suggests that resistance exercise is the intervention that shows a positive effect on muscle fat mass, and bone mass. More research is needed for other exercise interventions.

Globally, a demographic transition process is being experienced [1]. According to the World Health Organization (WHO), projections for 2050 suggest that 60% of the population in Europe will be 60 years or older, while in North America and Latin-America and the Caribbean, the percentage will be approximately 28% and 25% respectively [2]. This accelerated process poses new challenges for the design of health policies and strategies for this population. Older adults (OA) are a vulnerable group; therefore, it is necessary to generate scientific evidence that helps improve their quality of life and promote healthy aging. Developing and maintaining functional abilities that allows well-being in older age is a process defined by WHO (2015) as healthy aging [2].

Muscle mass, fat mass, and bone mass, muscular strength, and physical performance are three key elements in the health status of the older adults. They are strong predictors for different geriatric syndromes (GS), such as sarcopenia, osteoporosis, and its derivations [3]. For example, osteosarcopenic obesity (OSO) is characterized by alterations in three tissues: bone, muscle, and adipose tissue [4]. Ageing becomes one of the main risk factors for GS that are linked with worse conditions such as frailty, increased risk of fractures, physical disability, and longer hospital stays [5].

From 20-80 years muscle mass loss had been estimated 35-40%, also a difference of 20-40% between young and OA in muscle strength and increases more than 50% in adults over 80 years [6]. After menopause, there is an increase of body weight and fat mass percentage as a result of incrementation in visceral fat and diminished fat-free mass. In men, testosterone promotes muscle regeneration through the activation of satellite cells; testosterone decreases approximately 1% per year, which can negatively affect the distribution of muscle mass and fat mass aging [7]. The hormonal axis, including the somatotropic axis (GH/Insulin-like growth factor-1 IGF-1) related to protein metabolism, the rate of muscle excitation and contraction is associated to reduce their activity with increasing age; therefore, the ratio of muscle quality has a significant decrease associated with age [8,9]. After 50 years old, the average annual bone loss is 1-2% per year. In total, one of three women in this life stage experience osteoporotic fractures, while in men it occurs in one of five [10]. The loss of muscle mass is accompanied by a greater decline in muscle strength and power that can lead to accelerated muscle atrophy, resulting in increased risk of physical disability [11].

Physical performance has been defined as an objective measurement of the body’s function and locomotion [12]. Some physiological changes such as loss of motor units, changes in fiber types, atrophy of muscle fibers, and reduction of neuromuscular activation can affect the speed, strength, and power of movements leading to a reduction in physical performance [13]. It is important to include other components as fat mass, bone mass and muscle mass to have a better insight when comparing functional and strength measures, the gap linking muscle mass, strength and functionality will reduce [3].

Previous studies have suggested that exercise has an important effect on the functionality and body composition of older adults [13,14]. For example, Cruz-Jentoft *et al.* have concluded that strength and resistance exercise can improve the physical performance of senior adults, specifically in chair lifting tests, 12-minute walks and stair climbing. Mixed strength and aerobic exercise show an improvement in muscle strength, however, no increases in muscle mass were observed in either case [15]. Steffl *et al.* (2017) observed that physical activity has a protective affect against the development of sarcopenia [16].

Thus, the aim of this systematic review is to assess the effect of exercise on muscle mass, fat mass, bone mineral content, strength, and physical performance for community dwelling older adults.

## MATERIALS AND METHODS

This systematic review followed a methodology guided by the Cochrane handbook for systematic reviews of interventions [17]. The protocol was published at PROSPERO, ID: CRD42020207148.

### Criteria for include studies in this review

#### Type of studies

The studies included were randomized clinical trials (RCTs), without language restrictions, from January 2010 to October 2021. We decided to make a cut from almost ten years back in the literature, taking in consideration that the evidence is in constant renovation.

#### Types of participants

The participants considered for this review were healthy aged population, adults older of 60 years, who lived in a community with sarcopenia or pre-sarcopenia o diminished muscle mass. The exclusion criteria were clinical trials where the participants were living in hospitals or were institutionalized, participants with health complications as cancer, heart failure, kidney failure, diabetes and in recovery of post-acute illness or post-surgery.

#### Types of interventions

Were included all kind of exercise, trials comparing exercise to control group or other exercise group. As exclusion criteria, were excluded trials that combined exercise intervention with nutrition supplementation or specific diet, and electrostimulation.

#### Types of outcomes

Major outcomes: Muscle mass, fat mass and bone mass, measured by dual-energy X-ray absorptiometry (DXA), bioimpedance (BIA) or tomography computerized (CT).

Secondary outcomes: Physical performance and muscle strength.

#### Electronic searches

A search strategy was designed for the following databases: PubMed Medline, LILACS, Trip database, Epistemonikos, SPORTDiscus, PEDro, Scopus, Web of Science, SciELO, Cochrane library and Science Direct. The search strategy was designed using validated MeSH terms and decided by consensus of the authors. MeSH terms as “aged”, “sarcopenia”, “exercise”, and “obesity”; other terms used were “elderly”, “sarcopenic obesity”, “osteosarcopenic obesity” and “physical activity” were used. The full search strategy used per database was described in Supplementary Table 1. The databases used were mostly with artificial intelligence that helped to mix these terms with similar terms.

**Table 1:** Characteristics of included studies of three types of exercise (resistance, aerobic and mixed) interventions on community-dwelling older adults.

Author & year		N	Sex (F, %)	Type of exercise	Time of intervention (per week)	Intervention	Main findings
**Kim et al., 2012 [63]**	Exercise group	36	100%	Resistance	12 weeks (2)	60 minutes: 5-minute warm- up, 30 minutes of strength exercise, 20 minutes of balance and gait training, and 5 minutes of cool down. 12-14 on the Borg Rate.	↑ Usual and maximum speed walking
	Health education group	37	100%	NA		Health education brochures.	
**Kim et al., 2013 [30]**	Exercise group	30	100%	Resistance	12 weeks (2)	60 minutes: 5-minute warm- up, 30 minutes of strengthening exercise, 20 minutes of balance and gait training, and 5 minutes of cool down. 12-14 on the Borg Rate	↑ Usual walking. No significant differences in body composition
	Health education group	28	100%	NA		Health education brochures.	
**Balachandran et al., 2014 19]**	Power circuit training (HSC)	8	100%	Resistance	15 weeks (2)	40-45min. 3 series of 10-12 repetitions no recovery until complete 11 exercise, then 1-2 min of recovery. 50-60% de 1RM	Both groups: ↑SPPB and gait speed, leg press peak power, lower body strength. %FM, SMI improve but no differences between groups. ↑HSC group: sit-to-stand, pan carry, physical function.
	Strength/hypertrophy training (SH)	9	88%	Resistance	15 weeks (2)	55-60 min: 11 exercise, 3 series of 10-12 repetitions 1-2 min of recovery. 70% of 1RM, Progressive increasing 5%.	
**Watanabe et al., 2015 [44)**	Resistance exercise slow movement and tonic force generation	20	50.0%	Resistance	16 weeks (2)	20-30 min: five resistance exercise and four light plyometric exercise. 3-s concentric, 3-s eccentric and 1-s isometric)	No changes in body composition and physical function between groups. ↑ LSTM. Significant time effects for all strengths of the upper and lower limbs, two-step value, maximum leg extensor power. ↓%FM
	Resistance exercise normal speed	19	50.0%	Resistance	16 weeks (2)	20-30 min: five resistance exercise and four light plyometric exercise. 1-s concentric, 1-s eccentric and 1-s rest)	
**Chang et al., 2016 [22]**	Experimental group	42	88.1%	Mixed	12 weeks (3)	30 min: Qigong eight-form moving meditation	Medium-to-large effect size fat and lean mass, functional performance, HGS, lower limb muscle strength, and power.
	Control group	48	64.6%	NA		No intervention	
**Hong et al., 2016 [26]**	Teleexercise group	11	54.5%	Resistance	12 weeks (3)	20 min progress to 40 min. 8 exercise week 1-4 no weight, week 5-8 weight of 1 kg, week 9-12 weight 2kg. 3 series of 8-10 repetitions, < 1min of recovery.	Significant interaction effect between group and time on lower limb mass, ASM and TSM. ↑ Chair stand and chair sit-reach
	Control group	12	63.6%	NA		No intervention	
**Kim et al., 2016**	Exercise group	35	100.0%	Mixed	12 weeks (2)	60 minutes: Warming + resistance exercise seated to stand, elastic resistance bands, machine 1 to 3 sets of 10 repetitions +stationary bicycle aerobic exercise, and chair/standing exercise 12 minutes for cooling.	↓Trunk fat mass and ASM. ↑Stride, step length and knee extension
	Control group	34	100.0%	NA		Health education	
**Maruya et al. 2016**	Intervention group	26	55.9%	Mixed	6 months (2)	20-30minutes of walking, 3 sets of 6 repetitions de squats, 3 sets of 20 repetitions heel lift, balance with 1 leg raised for 1 minute.	↑ handgrip strength, knee extension strength. ↑ (lightly) SMI. ↓ %FM
	Control group	14	55.6%	NA		No intervention	
**Gadehla et al. 2016**	Exercise group	69	100%	Resistance	24 weeks (3)	8 exercises, 3 sets, progressive intensity increase, with training loads equal to 60% of 1-RM first four weeks, 70% following four weeks, and 80% remaining 16 weeks, with repetitions respectively decreased from 12, 10 and 8.	↑ FFM and appendicular FFM, ↓body fat. ↑ 1RM values
	Control group	64	100%	NA		No intervention	
**Marcin et al., 2016**	Experimental group	22	100%	Aerobic	12 weeks (3)	60 minutes: 10 min warming up, 40 Nordic walking, 10 min cool down	↑ SMI and SM. ↓%FM. ↑knee extensor ↑ TUG and 6MWD
	Control group	23	100%	NA		No intervention	
**Chen et al., 2017**	Aerobic training	15	93.3%	Aerobic	8 weeks (2)	60 minutes: 5-10 minutes of dynamic stretching and warm up and 40-45 minutes of the actual training.	↑SMM and Muscle Mass Index*. ↓ %FM and VFA. ↑ grip strength
	Resistance training	15	80.0%	Resistance	8 weeks (2)	60 minutes:10 exercise, 3 sets of 8-12 repetitions, 2-3minute rest between sets. Progressive intensity 60-70% 1RM	↓%FM and VFA, ↑ muscle mass index*. ↑grip strength, back extensor and knee extensor.
	Combination training	15	73.3%	Mixed	8 weeks (2)	1-day aerobic training and 1-day resistance training. Previous decribe.	↑ SMM and muscle mass index. ↓%FM and VFA. ↑ grip strength
	Control group	15	86.6%	NA		No intervention	
**Huang et al., 2017**	Elastic band resistance training	18	100.0%	Resistance	12 weeks (3)	55min; 10 min warming + 40min Resistance strength with elastic band + 5minutes cooling. 10 exercise, 3 series of 10 repetitions. Vigorous to 13 Borg RPE scale	↓%FM right upper extremity, left upper extremity, TFM, %FM. ↑BMD, T-score, Z-score.
	Control group	17	100.0%	NA		No intervention	
**Liao et al., 2017**	Experimental group	25	100.0%	Resistance	12 weeks (3)	60 min: 10min warming + 35-40min resistance exercise 3 series of 10 repetitions (6 exercise different muscle groups) + cool down.	↓Fat-free mass. ↑ MQ, and physical capacity. ↓Sarcopenia and physical difficulty
	Control group	21	100.0%	NA		No intervention	
**Park et al., 2017**	Exercise group	25	100.0%	Mixed	24 weeks (5)	50-80 min aerobic: 30-50 min walking, 5 days, 13-17 Borg scale + resistance exercise: 3 days 20-30min elastic band, 8-15 repetitions, 2-3 sets.	↓%FM, WC, SBP. ↑ chair stand-up, sit- and-reach, 2-min walking.
	Control group	25	100.0%	NA		No intervention	
**Caravalho et al., 2018**	OB-DY1	21	0.0%	Resistance	12 weeks (3)	10-min warm-up + 4 exercises, 3 sets, 12 repetitions 80% 1RM + 6 functional exercises, 3 sets, 10 repetitions 10 Borg scale+ 10-min stretching exercises. 80% 1RM	Medium-to-large effect size fat and lean mass, functional performance, HGS, lower limb muscle strength, and power.
	OB-DY2	18	0.0%	Resistance	13 weeks (3)	10-min warm-up + 4 exercises, 3 sets, 12 repetitions 80% 1RM + 6 functional exercises, 3 sets, 10 repetitions 10 Borg scale+ 10-min stretching exercises. 80% 1RM	
**Chen et al., 2018**	Kettlebell training	17	100.0%	Resistance	8 weeks (2)	60 minutes:11 exercises, 8-12 repetitions, 3 sets. 60-70% 1RM	↑ ASM and sarcopenia indexα. Significant two-way interaction of groups and time for SMM, ASM, sarcopenic index. ↓VFA ↑LHG, RHG, BS.
	Control group	16	100.0%	NA		No intervention	
**Liao et al., 2017**	Elastic band resistance training	33	100.0%	Resistance	12 weeks (3)	55 minutes: 10-min warm-up, 40-min period of elastic resistance exercises, and 5-min cool-down. Progressive increasing to 13 Borgs RPE scale.	↑ASM, Sarcopenia index, absolute muscle mass. ↑ muscle quality (MQ), and Physical function, gait speed, single leg stance (SLS) test, TCR. ↓ (TUG) test and timed chair rise (TCR).
	Control group	23	100.0%	NA		No intervention	
**Tsekoura et al., 2018**	Group based	18	89.0%	Mixed	12 weeks (3)	100 min (weekly) walking divided in 3 + 60 min progressive strength: 5-10 min warm-up. 20-30-min of strengthening, 20 min of balance and gait training, 5-10 min cool-down. In group	↑SMMI, TUG Handgrip strength (kg) Gait speed, 4 m test, chair stand test. Quality of life (SarQoL) Calf circumference (cm) and limb strength.
	Home based	18	84.0%	Mixed	12 weeks (3)	100 min (weekly) walking divided in 3 + 60 min progressive strength: 5-10 min warm-up. 20-30-min of strengthening, 20 min of balance and gait training, 5-10 min cool-down. With a physioterapist in their home.	↑TUG, Gait Speed, 4 m test, CS test, QOL, knee muscle strength-right knee flexion 180°/s, right knee extension 90°/s, and left knee flexion 180°/s.
	Control group	18	89.0%	NA			
**Cunha et al., 2017**	1 set group (G1S)	21	100.0%	Resistance	12 weeks (3)	30 minutes: 1 set of 10-15 repetitions. 8 exercise 2-3 min recovery.	↑ G3S higher strength changes than G1S. ↓%FM G3S compared to G1S. ↑ no difference between groups. ↑ BMD z-score G3S
	3 sets group (G·S)	20	100.0%	Resistance	12 weeks (3)	50 minutes: 3 set de 10-15 repetitions. 8 exercises. 2-3min de recovery	
	Control group	21	100.0%	NA		No intervention	
**Zhu et al., 2018**	Exercise group	40	72.5%	Mixed	12 weeks (3)	45 minutes: 5-10 min warm up and cool down + 20-30 min resistance Therabands + 20-min aerobic exercises. 8 exercise for upper and lower extremities, 6 sets, 6-8 repetitions. 1 day at home exercises with the resistance bands. 40% of 1 RM and progressive increase. Progressive change of the resistance of the bands.	↑leg extension, five-chair stand test, and physical activity level. No significant changes in body composition
	Control group	37	78.4%	NA		No intervention	
**Jung et al., 2019**	Exercise group	13	100.0%	Aerobic	12 weeks (3)	25-75-minute: 10 movements + 10 min cold down.	↓%FM, FFM. ↑Balance, 10m walking and peak power.
	Control group	13	100.0%	NA		No intervention	
**Vezzoli et al., 2019**	SAR-RT	20	50.0%	Resistance	12 weeks (3)	6-8 min aerobic warm-up + 3 series, 14-16 repetitions, 4 exercises, 60% 1RM.	↑Muscle strength. No differences in body composition. ↑stair climbing
	SAR-NT	15	60.0%	NA		No intervention	
**Yamada et al., 2019**	Exercise group	28	18.0%	Resistance	12 weeks (2)	30minutes: 5 min warm-up activity + 20 min resistance exercise program + 5 min of cool-down. 7 exercises, 3 sets, 20 repetitions	↑maximum walking speed, knee extension. No significant changes in body composition.
	Control group	28	15.0%	NA		No intervention	
**Banitalebi et al., 2020**	Exercise group	32	100.0%	Resistance	12 weeks (3)	80 min: Warm-up 10 min + resistance training session (60 min), 1-2 exercises (in a slow controlled manner, 2 s for concentric phase and 4 s for eccentric phase) + cool-down routine.	No significant changes in body composition. ↑lightly BMD, BMC. ↑HGS, 30s chair stand.
	Control group	31	100.0%	NA		No intervention	
**Makizako et al., 2020**	Exercise training group	36	72.2%	Mixed	12 weeks (1)	60 minutes: Warm-up involving stretching + 25 to 30 min resistance training, 10 exercises, progressive intensity +20 to 25 min balance + aerobic exercises + 5 min cool-down. 12-14 Borg scale. Daily home base exercise.	↑ maximum gait speed, chair stand, TUG test. No changes in muscle mass
	Control group	36	69.4%	NA		No intervention	
**Jeon et al., 2020**	Experimental group	13	100.0%	Mixed	12 weeks (5)	30 minutes: 5 min warm-up + 5 min upper body resistance + 5 min lower body resistance + 5 min aerobic + 5min flexibility + 5 min cool-down. Augmented-reality UINCARE-HEALTH tm. 9-13 Borg scale	↑ ASM, SMI. ↑Chair stand, TUG, 2-min step, gait speed.
	Control group	14	100.0%	NA		No intervention	
**Morawin et al., 2021**	Tai-Chi training	27	NR	Resistance	16 weeks (2)	Yang-style 24-form Tai-Chi training program	Body composition no significant changes. ↑HGS, gait speed
	Health education	36	NR	NA	16 weeks (2)	Health education	
**Seo et al., 2021**	Resistance intervention	13	100.0%	Resistance	16 weeks (3)	60 minutes: 5 min warm up + 50 min resistance training with elastic bands, 11 exercises upper body and 7 exercises lower body, progressive 6-15 repetitions, 3-5 sets + 5 min cool down	↑ Functional fitness, grip strength, gait speed, and isometric muscle strength.
	Control group	10	100.0%	NA		No intervention	
**Lee et al., 2021**	peRET group	15	100.0%	Resistance	12 weeks (3)	60 min: 10 min of warm-up exercises + 40 min of elastic band resistance exercises, 5 muscle groups 1-2 exercises, 3 sets, 10 repetitions + 5 min of cooling-down.	↑BF%, TSM, and LMI. GS, TUG, TCR.
	Control group	12	100.0%	NA		No intervention	

#### Reference list scanning

The reference list of other reviews related to our topic in terms of interventions and outcomes and the reference list of the studies included in this review were examined.

#### Selection stage

**Figure 1.**
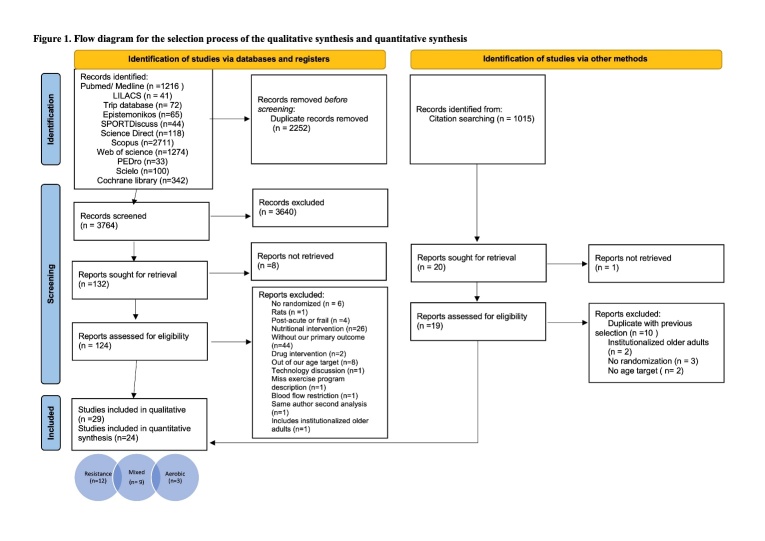
Flow diagram for the selection process of the qualitative synthesis and quantitative synthesis.

Two authors of this review independently screened titles and abstracts (AG-R and ED-G) with the objective of screen all the relevant articles. Then for selection process, reviews were removed, full texts of the remaining articles were retrieved and finally, those were systematically examined for inclusion or exclusion. Then, other authors (AG-R and LM-S) identified the articles with comparable methods for quantitative analysis. In the event of disagreement, a third author decided whether or not to include the article (LM-S for the screening and ED-G for the quantitative identification). The selection stages are found in the Figure 1.

### Data extraction and management

The information was extracted based on the *PICO* question, beginning by identifying the initial characteristics of the *Population*, such as age, sex and size of the sample studied. For *Intervention* and *Comparison*, characteristics of the type of intervention were organized according to frequency, intensity, type of exercise, time and progression of exercise, time of the intervention, and time of follow-up. We used categories to divide in types of exercise as: resistance exercise, aerobic exercise, and mixed exercise (aerobic + resistance exercise). The *Outcomes* were organized by changes in body composition, seeking to identify the changes in muscle mass (total and appendicular), fat mass (percentage and in grams), and bone mass (bone mineral density [BMD]). Changes in physical performance (gait speed, short physical performance battery [SPPB], Timed up and Go test [TUG]) and in muscular strength (grip strength and chair lift) were evaluated.

### Risk of bias assessment

Two reviewers independently assessed the risk of bias for each study using the criteria outlined in the *Cochrane Handbook for Systematic Reviews of Interventions* [17]: 1) random sequence generation (selection bias), 2) allocation concealment (selection bias), 3) blinding of participants and personnel (performance bias), 4) blinding of outcome assessment (detection bias), 5) incomplete outcome data (attrition bias), 6) selective outcome reporting (reporting bias) and, 7) other bias.

Each potential bias was graded as high, low or unclear risk. Risk of bias was evaluated by AG-R and LM-S.

### Publication bias

A funnel plot was performed with Review Manager 5.4 software, for the outcomes we had enough data.

### Measurement of treatment effect

For the meta-analysis were included RCTs with a control group and comparable measures. An additional were made when enough and comparable data were available. The effect of the evaluated interventions included: mean difference and standard deviation. An analysis model was used according to heterogeneity, when I^2^ ≥50% a randomized effect model was used and when I^2^ <50% a fixed effect model (18). All analyses were performed with Review Manager 5.4 software. Similar intervention and temporality characteristics were identified for statistical comparisons between studies.

## RESULTS

### Search results

The results of the screening process of the studies are shown in the PRISMA flowchart (Fig. 1). In total, we identified 7,031 records; of these 5,711 were obtained with the previously mentioned search strategy and 1,015 thru scanning reference list of similar reviews and included studies. From the databases search, we eliminated 2,252 duplicate studies, we screened 3,764 by title and abstract, excluded 3,640 that did not meet the inclusion criteria. For example, observational studies, studies in animals or studies of older adults with cancer. Then, 132 reports were sought for retrieval, eight studies were not retrieval because they were not fully published. Then, 124 full-text articles were analyzed. Of those, 96 articles did not meet the inclusion criteria and were eliminated, of the excluded studies: Six were not randomized, one was on rats, four were with frail or post-acute participants, 26 had a nutritional intervention (i.e. use of nutritional supplements), 44 have not information about our primary outcome (body composition), two had a drug intervention, eight had participants out of our target of age (i.e. postmenopausal women from 50 years or more), other reasons were studies about technology discussion, missing exercise program description and includes institutionalized older adults. Finally, 29 studies [19-47] were included in the qualitative analysis and 24 in the quantitative analysis. Four studies [19,21,43,44] that had just intervention groups and one [25] with data in percentage of change were excluded of quantitative analysis as they were not comparable with the other studies.

### Included Studies

The main characteristics of the primary studies are described in Table 1.

All the studies were RCTs. In 15 studies [20,25,27-34, 39,41,48] participants were only women, 13 studies [19,21-23,26,36,37,40,42,43,46,49,50] had participants of both genders and one [38] did not report the gender of the participants. In terms of the place where the studies were carried out, seven studies were carried out in Taiwan [22,23,27,33-35,47], seven in Japan [30-32,36,37,44,50], five in Korea [26,28,29,40,41], two in Brazil [25,48], two in Poland [38,39] and only one in the United States of America (USA) [19], China [46], Italy [43], Canada [21], Iran [20], and Greece [42].

Regarding the three types of interventions categorized, 19 studies had a resistance-type exercise intervention [19-21,23,25-27,30-35,38,41,43,44,47,48, 50], three studies had an aerobic-type intervention [23,29,39], and nine had a mixed intervention (resistance + aerobic exercise) [22,23,28,31,36,37,40,42,46]. Regarding the duration of the intervention for the resistance exercise intervention, 12 studies their intervention lasted 12 weeks [26,27,31,34,39,42,46,48], in three studies lasted less than 12 weeks [23,47,51], and in four studies it lasted longer than 12 weeks [19, 25,37,52]. Regarding the number of sessions, the mode was three sessions of physical exercise per week. For aerobic exercise, two studies [29,39] were by 12 weeks, three sessions per week and one study [23] was by eight weeks, two times per week. For mixed exercise six studies [22,28,31,36,42,46] had the duration of 12 weeks, 24 weeks for two studies [37,40] and eight weeks for one study[23], the range of sessions per weeks was one to five sessions per week, three studies had two sessions per week.

### Risk of Bias assessment

The risk of bias graph and summary can be found in the Supplementary Figure 1.

A high risk in other biases was also identified; for example: selection bias, since in several of the selected studies the participants enrolled in the clinical trial by their own initiative, which could suggest that these OA are more interested in exercise and taking care of their health.

### Random sequence generation (selection bias)

Six studies [22,23,26,39,42,47] are at unclear risk for not specifying the method in which the random sequence was generated.

### Allocation concealment (selection bias)

Nine studies [19,22,23,26,37,39,41,47,49] have an unclear risk as they did not report the method used to conceal allocation between groups. Tsekoura *et al.* [42] are at high risk because the participants were given envelopes and they could easily see each other’s allocations.

### Blinding of participants and personnel (performance bias)

A total of 11 studies [21,25,30,32,34-36,38,42,43,46,47] have unclear risk. For example, Liao et al. (2017) reported blinding of the participants; however, they also notified the participants of the types of interventions by informed consent had low risk. Yamada et al. (2019) had low risk of bias because they report blinding for the participants and for the personnel. A total of 17 studies [20,22,23,26-29,31,33,37,39-41,44,48] have a high risk of bias due to the nature of the intervention, because it is difficult to keep participants blinded as they had to know the intervention and possible adverse effects.

### Blinding of outcome assessment (detection bias)

Fifteen studies [21,22,25,26,28,29,31,37-40,43,47,48,50] had an unclear risk as they did not report clearly blinding.

### Incomplete outcome data (attrition bias)

Six studies [25,32,34,39,47,49] have unclear risk because they do not report this. Two studies [37,46] have a high risk of bias because the loss of participants affects the balance of the randomized groups.

### Selective outcome reporting (reporting bias)

Twelve studies [19,23,26,29,31,34-37,43,47,48] have unclear risk, mainly because they did not report or publish their protocol.

### Effects of interventions

Studies were identified to had comparable data for the quantitative analysis, these studies were classified by type of exercise and unit of measure. In this sense, 13 studies [20,23,26,27,32-35,38,41,47,48,50] were identified from resistance exercise intervention, three studies [23,29,39] for aerobic intervention and eight [22,23,28,31,36,37, 40, 42,46] for mixed exercise.

### The certainty of evidence

The complete GRADE Summary of Findings table for all the comparisons are reported in Supplementary Table 2. The certainty of evidence was evaluated in the comparison of resistance exercise vs. control group for four outcomes with high certainty: 1) total muscle mass (Kg), 2) appendicular muscle mass, 3) total body fat mass (kg), 4) body fat percentage (%). In the same comparison bone mineral density had moderate certainty. For aerobic exercise compared to control group the outcome fat mass percentage (%) shows high certainty. For mixed exercise, compared to control group for muscle mass index (kg/m^2^) moderate certainty, fat mass percentage (%) presents high certainty. Physical performance and strength comparison for resistance exercise and mixed exercise are reported in Supplementary table 1. Finally, aerobic exercise had not comparable data for the secondary outcomes.

**Figure 2.**
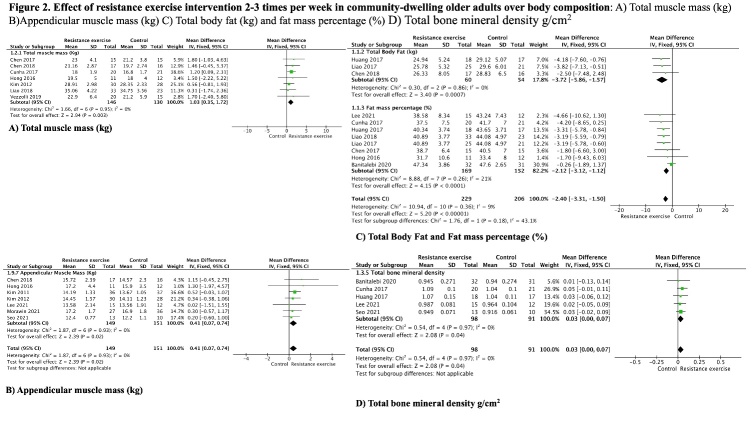
**Effect of resistance exercise intervention in community-dwelling older adults over body composition**. A) Total muscle mass (kg). B) Appendicular muscle mass (kg). C) Total body fat (kg) and Fat mass percentage (%). D) Total bone mineral density g/cm^2^.

### Resistance exercise interventions

Considering our primary outcome and resistance exercise, we compared seven studies [23,26,30,35,43,47,48] for muscle mass, measured by total muscle mass (Kg) (Fig. 2A) with 146 total participants in the intervention 2-3 times per week compared to 130 older adults in the control group, had an increase of 1.0kg (95%CI 0.3-1.7), heterogeneity of I^2^ = 0%. In another analysis, about appendicular muscle mass (ASM), seven studies [26,30,32,33,38,41,47] were included, with a total of 184 participants in the intervention and 185 in control group, heterogeneity of I^2^ = 0%, showed an increase of 0.4 kg (CI 95%: 0.07, 0.74) (Fig. 2B).

We evaluated three studies [27,34,47] with a total of 60 participants in the intervention and 54 in the control group that estimated the total fat mass (TFM) (kg) (Fig. 2C) and eight studies [20,23,26,27,33-35,48] with 169 participants in the intervention group and 152 participants in the control group estimating fat mass percentage (FM%) (Fig. 2C). This intervention results in a large reduction of 3.7 kg (95% CI: -5.8, -1.5) with heterogeneity of I^2^= 0% and a decrease of 2.1% (95% CI: -3.12, -1.12) with heterogeneity of I^2^= 21% respectively.

Findings in body composition measured by total BMD, were found in five studies [20,27,33,41,48] with a total of 98 participants and 91 older adults in control group. The intervention had a slightly increase of 0.03 (95%CI 0.00 - 0.07) with I^2^= 0% (Fig. 2D).

Evaluating strength, with handgrip strength resistance exercise results in a large increase 2.8 kg (CI 95%: 1.6, 4.0) in seven studies [23,30,33,34,38,43,47] with 149 participants (Supplementary Fig, 2A) and for timed chair rise, results in large increase 5.4 times (IC 95%: 4.16, 6.66) (Supplementary Fig. 2D) in 3 studies [26,34,35] with 69 participants and 56 control. In physical performance, TUG results in large reduction -2.06 seconds (Supplementary Fig. 2C) (IC 95%: -2.6, -1.5) in 5 studies [26,30,33-35], with 114 participants and I^2^= 0%, for gait speed the overall difference observed has no clinical relevance and is inconsistent (Supplementary Fig. 2B).

### Aerobic exercise interventions

**Figure 3.**
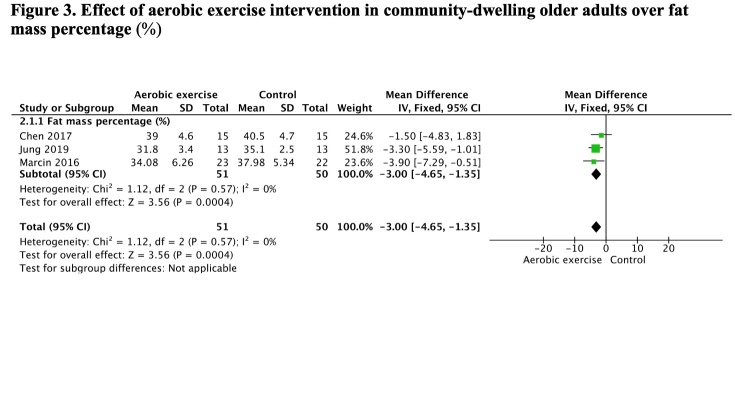
Effect of aerobic exercise intervention in community-dwelling older adults over fat mass percentage (%).

This meta-analysis was conducted of three studies [23,29,39] with 51 participants in the aerobic exercise intervention 2-3 times per week and 50 in the control by fixed effects model with heterogeneity of I^2^ = 0%. The results observed indicate a large reduction of -3.0% (CI 95%: -4.6, -1.3) in fat mass percentage (Fig. 3).

### Mixed exercise interventions

**Figure 4.**
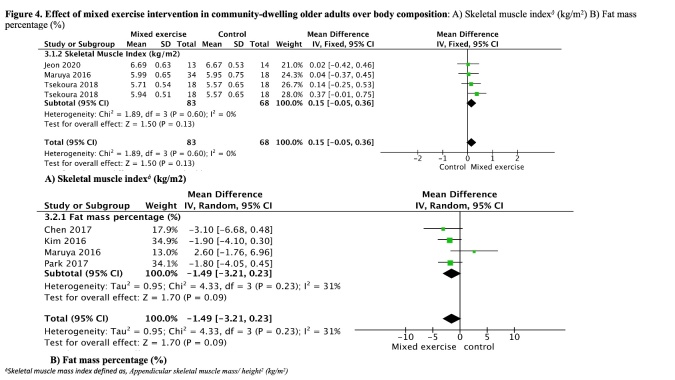
**Effect of mixed exercise intervention in community-dwelling older adults over body composition**. A) Skeletal muscle index*^å^* (kg/m^2^). B) Fat mass percentage (%).

Mixed exercise intervention results in little to no difference for muscle mass gain. The analysis of three studies [28,37,42]; including both intervention groups by Tsekoura *et al.* [42] with total of 83 participants receiving an intervention 2-3 times per week, with a heterogeneity I^2^=0% evaluated by the fixed effects model showed an increase of 0.1 (95%CI -0.0, 0.3). Also, were analyzed FM% for this intervention in four studies [23,31,37,40], with 101 participants receiving 2-3 times per week and 88 in the control group with a reduction by -1.4% (95% CI -3.2, 0.2), heterogeneity I^2^= 31% (Fig. 4). Both outcomes had not statistical significance.

For strength, were included six studies [23,28,31, 40,42,46] with 112 participants with 2-3 times per week intervention and 109 in control group evaluating handgrip, results in a slightly increase of 2.2kg (95% CI 0.0, 4.4) with heterogeneity I^2^ = 66%. For gait speed were included four studies [31,37,42,46] with 103 participants in the intervention 2-3 times per week and 83 participants in the control. They result in an increase of 0.08m/s (CI 95%: 0.03, 0.14) when compared with no exercise, with heterogeneity I^2^ = 17% (Supplementary Fig. 3).

Additionally, in the analysis by the total of sessions were found for total muscle mass an increase of 1.06 (95% CI: 0.15, 1.98) after 36 sessions (3 sessions per week). The detail of the additional analysis by total session are described in Supplementary Figure. 4.

### Publication bias

Funnel plots are presented in Supplementary Fig. 5. In this sense, no publication bias was detected.

## DISCUSSION

Improvements were observed after the resistance exercise interventions like, increase of total muscle mass, appendicular muscle mass, large decrease in total fat mass, and a decrease in body fat percentage. Regarding the aerobic exercise intervention, a decrease of body fat percentage was identified. For mixed exercise intervention, there was an increase in skeletal muscle mass index (SMI, defined by appendicular muscle mass kg / height^2^ m^2^), but the evidence is uncertain because there could be population with no effect, for FM% there was a slightly reduction but significant.

In the secondary outcome, for resistance exercise in physical performance were showed improvement in certain tests, such as the timed chair rise test with increased repetitions, the TUG test and gait speed with decreases in time, and for muscle strength was reported an increase in handgrip strength. In aerobic exercise for our secondary outcome of physical performance and strength, not enough information was obtained to perform a meta-analysis. With the mixed exercise interventions, there was an increase in gait speed and handgrip strength.

This review sought to analyze the effect of three types of interventions: resistance, aerobic, and mixed exercise, looking to primary outcomes like, muscle mass, fat mass, bone mass and secondary outcomes as, muscle strength and physical performance. In the final review, 19 studies had a resistance-type exercise intervention [19-21,23,25-27, 30-35,38,41,43,44,47,48,50], three studies had an aerobic-type intervention [23,29,39], and nine had a mixed intervention (resistance + aerobic exercise) [22,23, 28,31,36,37,40,42,46]. We identified training programs from eight to 24 weeks training, one to five sessions per week and 30 to 80min of workout.

The findings are similar to previous meta-analyses conducted in this population. According to another review, muscle mass increased in 82.8% of the studies (29/35 RCTs) following an exercise intervention, as well as an improvement in physical performance outcomes (26/28 RCTs, 92.8%) [53]. In this review, we identified an improvement in muscle mass after resistance exercise. In other metanalysis of seven studies Kim *et al.* [54] also shows a significant improvement in appendicular muscle mass, handgrip strength and TUG test, but the study only evaluated resistance exercise interventions. Bao *et al.* [55], identified that muscle strength (measured by timed chair rise test) and physical performance (measured by gait speed and TUG) had and improvement in 22 studies, but they did not stratify their results by type of exercise, also this study identified that they include institutionalized older adults and premenopausal women that had mean age below of 60 years old in their analysis. Otherwise, Escriche-Escuder et al, in their systematic review and meta-analysis did not found effect on muscle mass, by their inclusion criteria they analyze studies that used the EWSOP criteria for sarcopenia diagnosis [56].

For instance, an analysis with 47 studies representing 1079 participants suggests a significant association between resistance exercise and upper and lower body strength improvements among older individuals [57]. Other reviews suggest that exercise training programs may have beneficial effects on sarcopenic obesity-related parameters, such as body fat mass, muscle mass and strength [15,58]. In that review, all three studies assessing this parameter show that physical performance improved with resistance training alone versus the control group [58]. The present study reinforces that exercise for older adults is necessary and could have some benefits in different sarcopenia-related parameters.

Physical exercise has a positive impact on muscle mass and muscle function in older adults. The biggest effect of exercise intervention, of any type, has been seen on physical performance [53,59]; Lu et al, also identified that resistance training and mixed exercise suggest an improvement in TUG and Gait speed [60]. We identified a large increase in the “Chair rise test” and “TUG” after resistance exercise and an increase in gait speed after mixed exercise.

Aging is a process of constant changes, and a lack of activity further exacerbates the loss of muscle mass and function, resulting from obesity-derived inflammation [61]. This review is important to find a pathway to follow and help older adults to improve their muscle mass, physical performance, and muscle strength. The resistance exercise intervention helps activate the molecular regulation of muscle protein synthesis that is regulated by intrinsic cell signaling response [62]. Thus, its suggested that practicing resistance exercise and aerobic exercise will improve the utilization of energy and activation of molecular responses that will also improve muscle mass and decrease fat mass.

### Strengths and limitations

A comprehensive search for clinical trials was conducted; however, trials that did not clearly report the specific study population or intervention could have been omitted. In this sense, to minimize this possible bias, a systematic and objective process was followed for the search, for reporting results, and interpreting the evidence. The result of this analysis was evaluated on community dwelling older adults, so it could not be extrapolated to older adults in institutions or other conditions. One of the limitations in this analysis concerning the risk of bias is the absence or low quality of the blinding of the participants (60% high risk and 20% unclear risk). Although blinding can be complex in this type of intervention and could have an effect on the result. Search was conducted over the last ten years of evidence.

Three types of exercise were included in our study, a metanalysis for the different organized by comparable measures in different outcomes and we conducted the evaluation of certainty of the evidence.

Besides that, according to the literature research carried out for the present study, this is the first systematic review and meta-analysis focused on the exclusive effect of three types of exercise (resistance strength, aerobic and mixed) on muscle mass, body fat, bone mass, muscle strength and physical performance.

## Conclusions

Viewing our findings from a public health perspective, resistance exercise could treat and prevent age-related declines in muscle mass, bone mass, accumulation of fat mass, and physical performance. Additionally, is suggested at least 36 sessions with three sessions per week of resistance exercise for the gain of total muscle mass. Aerobic exercise may help to reduce the accumulation of fat mass. Future research with comparable units for muscle mass, bone mass and fat mass in needed for aerobic and mixed exercise.

## Availability of data and materials

The datasets used and/or analysed during the current study are available from the corresponding author on reasonable request.

## Supplementary Materials

The Supplementary data can be found online at: www.aginganddisease.org/EN/10.14336/AD.2021.0215.


