# Supplementary material for: Effect Of Exercise on Muscle Mass, Fat Mass, Bone Mass, Muscular Strength and Physical Performance in Community Dwelling Older Adults: Systematic Review and Meta-Analysis
Source: Aging Dis. 2022 Oct 1;13(5):1421–35. doi: 10.14336/AD.2022.0215 (PMC9466973; doi:10.14336/AD.2022.0215)
Supplement: Supplementary file 1 [file AD-13-5-1421-s.pdf]

SUPPLEMENTARY DATA

Effect Of Exercise on Muscle Mass, Fat Mass, Bone Mass, Muscular Strength and Physical Performance in Community Dwelling Older Adults: Systematic Review and Meta-Analysis

Alejandra González-Rocha[1,2], Lucia Mendez-Sanchez[3*], María Araceli Ortíz-Rodríguez[4], Edgar Denova-Gutiérrez[2*]

© 2022. González-Rocha A et al. Published online at http://www.aginganddisease.org/EN/10.14336/AD.2022.0215

SUPPLEMENTARY DATA

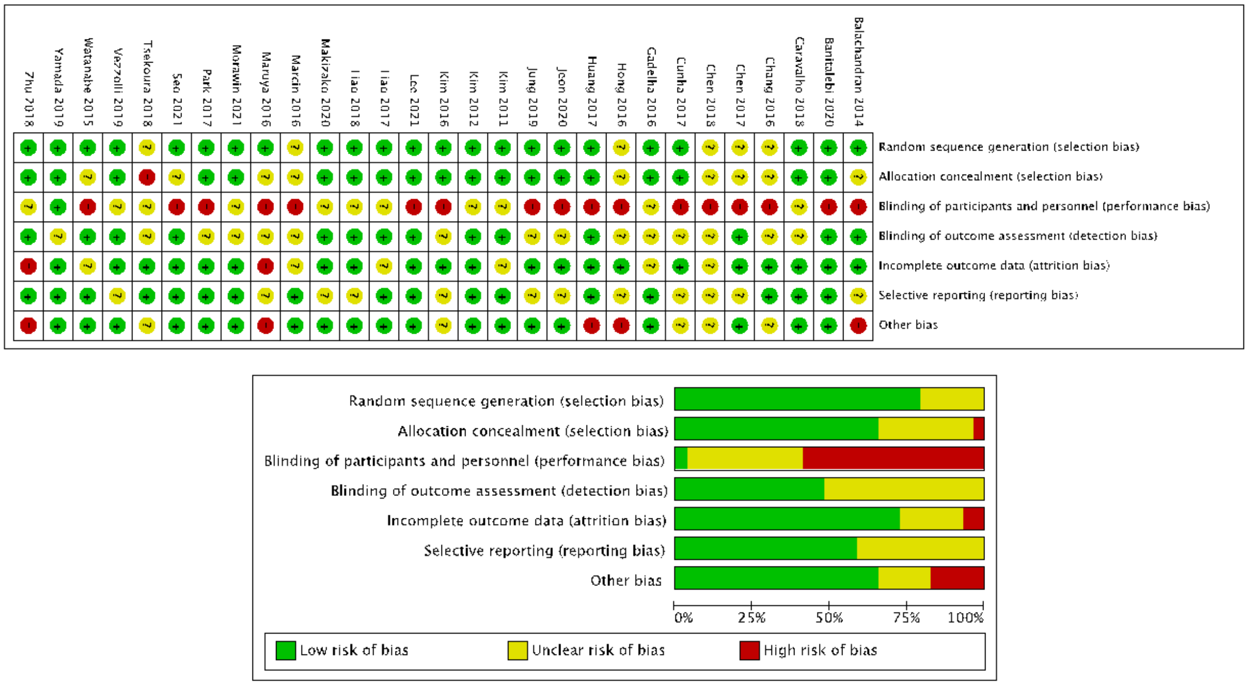

Supplementary Figure 1. Summary and graph of Risk of bias assessment.

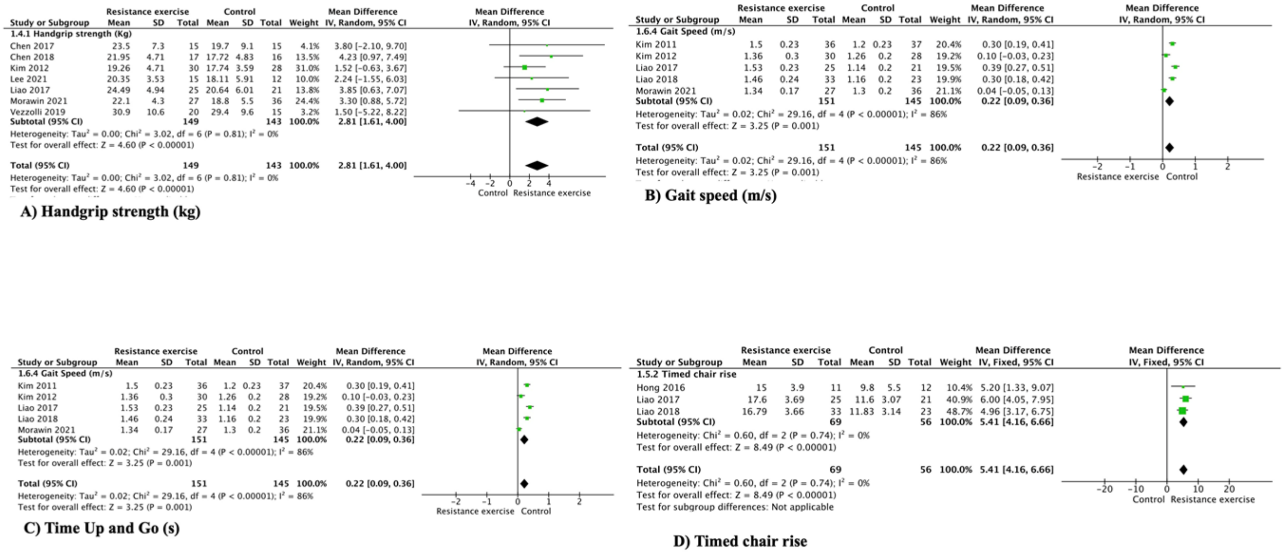

Supplementary Figure 2. Effect of resistance exercise intervention in community-dwelling older adults over muscle strength and physical performance. A) Handgrip strength (Kg) B) Gait speed (m/s) C) Time Up and Go (s) D) Timed chair rise.

© 2022. González-Rocha A et al. Published online at http://www.aginganddisease.org/EN/10.14336/AD.2022.0215

SUPPLEMENTARY DATA

Study or Subgroup	Mixed exercise Mean	SD	Total	Control Mean	SD	Total	Weight	Mean Difference IV, Random, 95% CI
**3.4.1 Handgrip strength (Kg)**								
Chen 2017	24.3	7	15	19.7	9.1	15	9.5%	4.60 [−1.21, 10.41]
Jeon 2020	22.91	6.16	13	22.37	8.37	14	10.2%	0.54 [−4.98, 6.06]
Kim 2016	20.3	3.8	35	21.1	4.1	34	23.7%	−0.80 [−2.67, 1.07]
Park 2017	26.9	2.5	15	22.8	2.8	15	23.6%	4.10 [2.20, 6.00]
Tsekoura 2018	20.58	4.29	18	17.92	4.14	18	19.7%	2.66 [−0.09, 5.41]
Zhu 2018	19.37	7.11	16	16.21	5.01	13	13.2%	3.16 [−1.26, 7.58]
**Subtotal (95% CI)**			**112**			**109**	**100.0%**	**2.21 [0.02, 4.41]**
Heterogeneity: $Tau^2 = 4.38$; $Chi^2 = 14.85$, df = 5 (P = 0.01); $I^2 = 66\%$								
Test for overall effect: Z = 1.98 (P = 0.05)								
**Total (95% CI)**			**112**			**109**	**100.0%**	**2.21 [0.02, 4.41]**
Heterogeneity: $Tau^2 = 4.38$; $Chi^2 = 14.85$, df = 5 (P = 0.01); $I^2 = 66\%$								
Test for overall effect: Z = 1.98 (P = 0.05)								

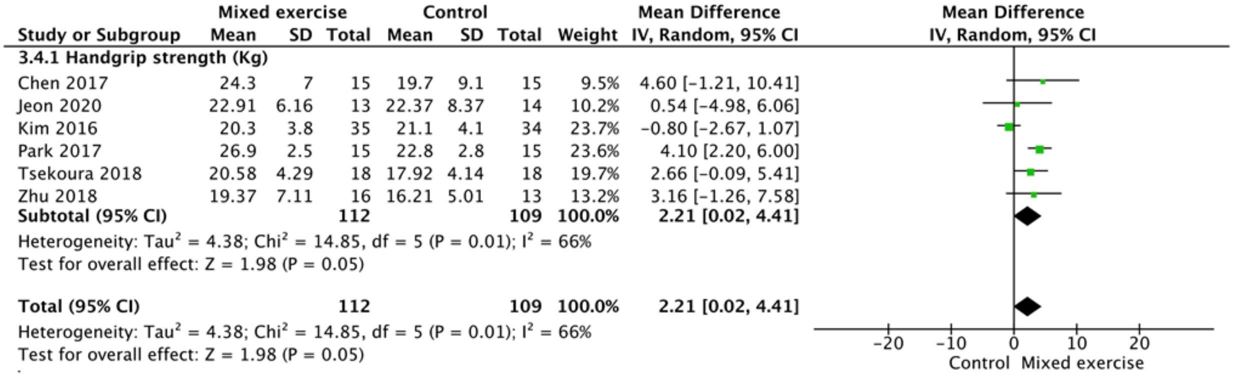

A) Handgrip strength (Kg)

Study or Subgroup	Mixed exercise Mean	SD	Total	Control Mean	SD	Total	Weight	Mean Difference IV, Fixed, 95% CI
**3.3.1 Speed Gait (m/s)**								
Kim 2016	1.3	0.2	35	1.2	0.2	34	33.9%	0.10 [0.01, 0.19]
Maruya 2016	1.46	0.2	34	1.42	0.17	18	28.2%	0.04 [−0.06, 0.14]
Tsekoura 2018	1.21	0.15	18	1.08	0.15	18	31.4%	0.13 [0.03, 0.23]
Zhu 2018	0.98	0.35	16	1.05	0.24	13	6.5%	−0.07 [−0.29, 0.15]
**Subtotal (95% CI)**			**103**			**83**	**100.0%**	**0.08 [0.03, 0.14]**
Heterogeneity: $Chi^2 = 3.61$, df = 3 (P = 0.31); $I^2 = 17\%$								
Test for overall effect: Z = 2.91 (P = 0.004)								
**Total (95% CI)**			**103**			**83**	**100.0%**	**0.08 [0.03, 0.14]**
Heterogeneity: $Chi^2 = 3.61$, df = 3 (P = 0.31); $I^2 = 17\%$								
Test for overall effect: Z = 2.91 (P = 0.004)								

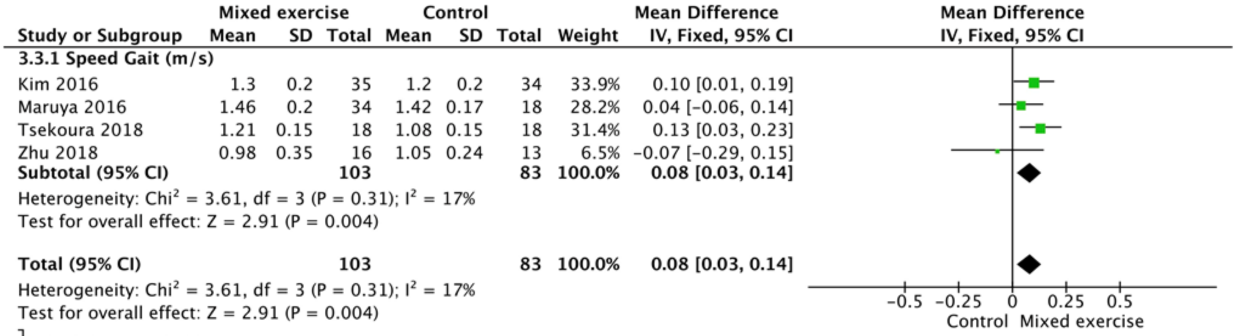

B) Gait speed (m/s)

Supplementary Figure 3. Effect of mixed exercise intervention in community-dwelling older adults over muscle strength and physical performance. A) Handgrip strength (Kg) B) Gait speed (m/s)

© 2022. González-Rocha A et al. Published online at http://www.aginganddisease.org/EN/10.14336/AD.2022.0215

SUPPLEMENTARY DATA

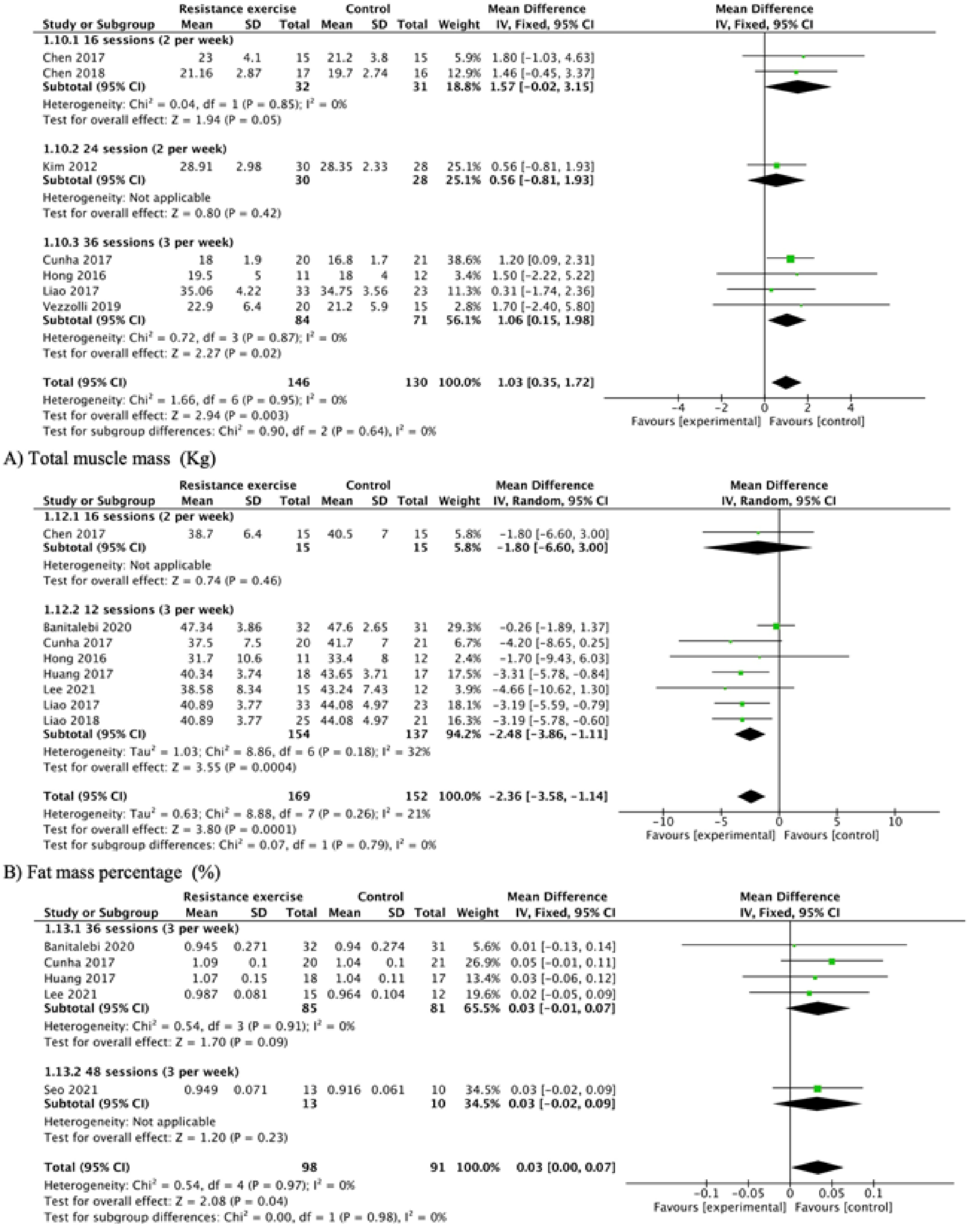

C) Total bone mineral density g/cm^2

Supplementary Figure 4. Effect of resistance exercise intervention in community-dwelling older adults over total muscle mass, appendicular muscle mass and fat mass percentage stratified by total number of sessions (sessions per week): A) Total muscle mass (Kg), B) fat mass percentage (%) C) total bone mineral density g/cm^2.

© 2022. González-Rocha A et al. Published online at http://www.aginganddisease.org/EN/10.14336/AD.2022.0215

SUPPLEMENTARY DATA

A) Resistance exercise intervention

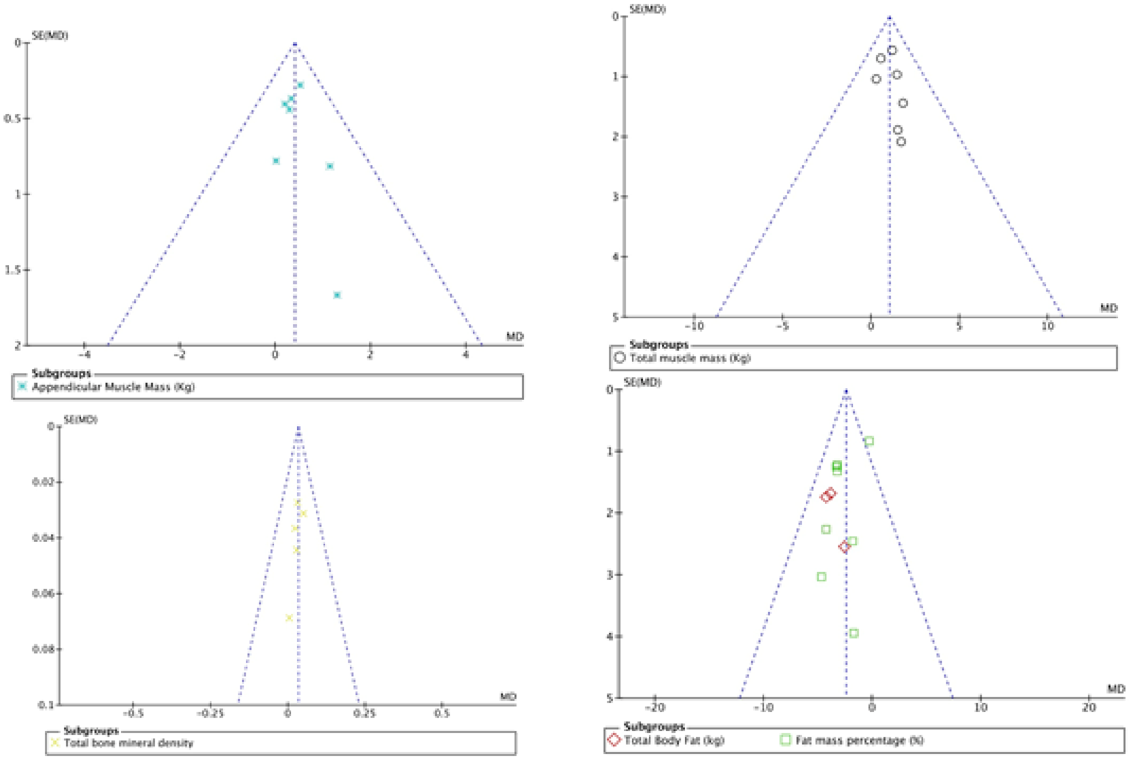

B) Aerobic exercise intervention

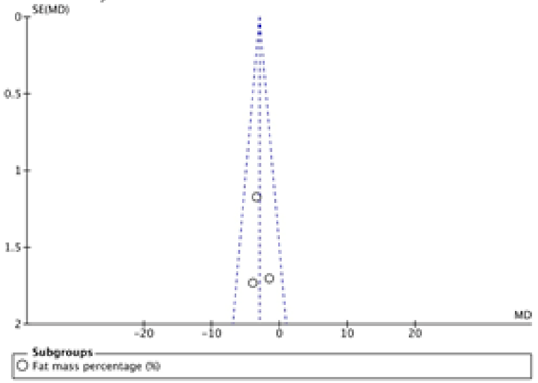

C) Resistance exercise intervention

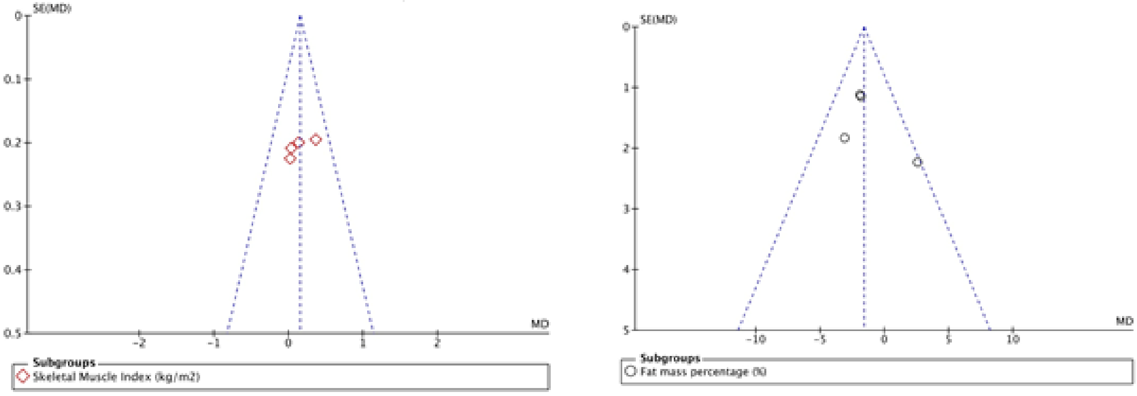

© 2022. González-Rocha A et al. Published online at http://www.aginganddisease.org/EN/10.14336/AD.2022.0215

SUPPLEMENTARY DATA

Supplementary Figure 5. Funnel plot of the primary outcome. A) Resistance exercise intervention, B) Aerobic exercise intervention, C) Mixed exercise intervention

Supplementary Table 1. Search strategy used per database.

Database	Terms	Limits	Search strategy
**Pubmed 1**	Sarcopenia AND aged AND exercise	Title/Abstract, 10 years	("sarcopenia"[Title/Abstract] AND "aged"[Title/Abstract]) AND "exercise"[Title/Abstract]
**Pubmed 2**	Osteosarcopenic Obesity AND aged AND exercise	Title/Abstract, 10 years	(("osteosarcopenic"[All Fields] AND ((((("obeses"[All Fields] OR "obesity"[MeSH Terms]) OR "obesity"[All Fields]) OR "obese"[All Fields]) OR "obesities"[All Fields]) OR "obesity s"[All Fields])) AND ("aged"[MeSH Terms] OR "aged"[All Fields])) AND (((((((((("exercise"[MeSH Terms] OR "exercise"[All Fields]) OR "exercises"[All Fields]) OR "exercise therapy"[MeSH Terms]) OR ("exercise"[All Fields] AND "therapy"[All Fields])) OR "exercise therapy"[All Fields]) OR "exercise s"[All Fields]) OR "exercised"[All Fields]) OR "exerciser"[All Fields]) OR "exercisers"[All Fields]) OR "exercising"[All Fields])
**Pubmed 3**	Sarcopenic Obesity AND aged AND exercise	Title/Abstract, 10 years	(("sarcopenic"[All Fields] AND ((((("obeses"[All Fields] OR "obesity"[MeSH Terms]) OR "obesity"[All Fields]) OR "obese"[All Fields]) OR "obesities"[All Fields]) OR "obesity s"[All Fields])) AND ("aged"[MeSH Terms] OR "aged"[All Fields])) AND (((((((((("exercise"[MeSH Terms] OR "exercise"[All Fields]) OR "exercises"[All Fields]) OR "exercise therapy"[MeSH Terms]) OR ("exercise"[All Fields] AND "therapy"[All Fields])) OR "exercise therapy"[All Fields]) OR "exercise s"[All Fields]) OR "exercised"[All Fields]) OR "exerciser"[All Fields]) OR "exercisers"[All Fields]) OR "exercising"[All Fields])
**Tripdatabase**	elderly with sarcopenia AND exercise		(title:elderly with sarcopenia)(title:exercise)
**Tripdatabase**	osteosarcopenic Obesity AND aged AND exercise		(osteosarcopenic obesity elderly)(exercise)
**Tripdatabase**	Elderly with sarcopenic obesity AND exercise		(title:elderly with sarcopenic obesity)(title:exercise)
**Epistemonikos**	Sarcopenia AND aged AND exercise	Title/Abstract, 10 years	(title:((title:(sarcopenia) OR abstract:(sarcopenia)) AND (title:(aged) OR abstract:(aged)) AND (title:(exercise) OR abstract:(exercise))) OR abstract:((title:(sarcopenia) OR abstract:(sarcopenia)) AND (title:(aged) OR abstract:(aged)) AND (title:(exercise) OR abstract:(exercise))))
**Epistemonikos**	Osteosarcopenic Obesity AND aged AND exercise	Title/Abstract	(title:((title:(osteosarcopenic obesity) OR abstract:(osteosarcopenic obesity)) AND (title:(aged) OR abstract:(aged)) AND (title:(exercise) OR abstract:(exercise))) OR abstract:((title:(osteosarcopenic obesity) OR abstract:(osteosarcopenic obesity)) AND (title:(aged) OR abstract:(aged)) AND (title:(exercise) OR abstract:(exercise))))
**Epistemonikos**	Sarcopenic Obesity AND aged AND exercise	Title/Abstract, 10 years	(title:((title:(sarcopenic obesity) OR abstract:(sarcopenic obesity)) AND (title:(aged) OR abstract:(aged)) AND (title:(exercise) OR abstract:(exercise))) OR abstract:((title:(sarcopenic obesity) OR abstract:(sarcopenic obesity)) AND (title:(aged) OR abstract:(aged)) AND (title:(exercise) OR abstract:(exercise))))
**SPORTDiscus s**	Sarcopenia AND aged AND exercise	Title, 10 years	TI Sarcopenia AND aged AND (exercise or physical activity)
**SPORTDiscus s**	osteosarcopenic Obesity AND aged AND exercise		TI Osteosarcopenic obesity AND Aged AND (exercise or physical activity)

SUPPLEMENTARY DATA

**SPORTDiscuss**	Sarcopenic Obesity AND aged AND exercise	Title, 10 years	TI Sarcopenic obesity AND Aged AND (exercise or physical activity)
**PeDRO 1**	Sarcopenia AND aged AND exercise	Title/Abstract	Sarcopenia AND aged AND exercise
**PeDRO 2**	Osteosarcopenic Obesity AND aged AND exercise	Title/Abstract	Osteosarcopenic obesity AND aged AND exercise
**PeDRO 3**	Sarcopenic Obesity AND aged AND exercise	Title/Abstract	Sarcopenia AND aged AND exercise
**Cochrane library**	Sarcopenic Obesity OR osteosarcopenic OR sarcopenia AND older adults AND exercise		older adults AND exercise AND (sarcopenia OR sarcopenic obesity OR osteosarcopenic)

Supplementary Table 2. Summary of Findings, GRADE certainty of evidence.

Outcomes	Anticipated absolute effects* (95% CI)		№ of participants (studies)	Certainty of the evidence (GRADE)	Comments
	Risk with Resistance exercise				
**Resistance exercise compared to control group for body composition, physical performance and strength**					
**Body Composition/ Muscle mass assessed with: Total Muscle Mass follow-up: range 8 weeks to 24 weeks**	mean 1.03 **kg more** (0.35 more to 1.72 more)	-	276 (7 RCTs)	⊕⊕⊕⊕ High	Resistance exercise results in a slight increase 1.03 kg (IC 95%: 0.35, 1.72) in Muscle Mass measured by total muscle mass. Heterogeneity I^2=0%
**Body composition - Muscle mass assessed with: Appendicular Muscle mass follow-up: range 8 weeks to 24 weeks**	mean **0.41 kg higher** (0.36 lower to 1.17 higher)	-	300 (7 RCTs)	⊕⊕⊕⊕ High	Resistance exercise increases in 0.41 kg (IC 95%: 0.07, 0.74) in Muscle mass slightly. Heterogeneity I^2=0%
**Body Composition Body Fat mass assessed with: Total Fat Mass follow-up: range 8 weeks to 24 weeks**	mean **3.72 Kg lower** (5.86 lower to 1.57 lower)	-	114 (3 RCTs)	⊕⊕⊕⊕ High	Resistance exercise results in large reduction of 3.72 kg (IC 95%: -5.86, -1.57) in Body Fat mass measured by Total Body Fat.
**Body Composition - Body fat assessed with: % Body Fat follow-up: range 8 weeks to 24 weeks**	mean **2.12% lower** (3.12 lower to 1.12 lower)	-	321 (8 RCTs)	⊕⊕⊕⊕ High	Resistance exercise results in large reduction of 2.12% (IC 95%: -3.12, -1.12) in Body Fat mass measured by Body Fat percentage. Heterogeneity I^2= 21%.
**Body Composition- Bone Mineral Density assessed with: Total Bone mineral Density follow-up: range 8 weeks to 24 weeks**	mean **0.03 g/cm2 higher** (0 to 0.07 higher)	-	189 (5 RCTs)	⊕⊕⊕◯ Moderate[a]	Resistance exercise 2-3 times per week intervention slightly increases Bone Mineral Density by 0.03 g/cm2 (IC 95%: 0.00, 0.07).
**Strength assessed with: Handgrip strength follow-up: range 8 weeks to 24 weeks**	mean **2.81 kg higher** (1.61 higher to 4.00 higher)	-	292 (7 RCTs)	⊕⊕⊕⊕ High	The evidence suggests resistance exercise results in a large increase 2.81 kg (IC 95%: 1.61, 4.00) in strength.
**Strength assessed with: Timed chair rise follow-up: range 8 weeks to 24 weeks**	mean **5.41 higher** (4.16 higher to 6.66 higher)	-	125 (3 RCTs)	⊕⊕⊕⊕ High	Resistance exercise results in large increase 5.41 times (IC 95%: 4.16, 6.66) in physical performance assessed with timed chair rise.

© 2022. González-Rocha A et al. Published online at http://www.aginganddisease.org/EN/10.14336/AD.2022.0215

SUPPLEMENTARY DATA

**Physical performance assessed with: Gait Speed follow-up: range 8 weeks to 24 weeks**	mean **0.22 m/s higher** (0.09 higher to 0.36 higher)	-	143 (3 RCTs)	⊕⊕◯◯ Low[a,b]	The overall difference observed has no clinical relevance and is inconsistent
**Physical performance assessed with: Time Up and Go follow-up: range 8 weeks to 24 weeks**	mean **2.06 s lower** (2.61 lower to 1.51 lower)	-	210 (5 RCTs)	⊕⊕⊕⊕ High	Resistance exercise results in large reduction -2.06 seconds (IC 95%: -2.61, -1.51) in physical performance measured by Time Up and Go test.
**Aerobic exercise compared to control for body composition, muscle strength and physical performance**					
**Body Composition - Fat mass assessed with: Fat mass percentage follow-up: range 8 weeks to 12**	MD 3.00 **lower** (4.65 lower to 1.35 lower)	-	101 (3 RCTs)	⊕⊕⊕⊕ High	Aerobic results in large reduction -3.00% (IC 95%: -4.65, -1.35) in body composition measured by fat mass percentage. Heterogeneity I^2= 0%
**Mixed exercise compared to control group for body composition, physical performance and strength**					
**Body Composition assessed with: Muscle mass index follow-up: range 8 weeks to 24 weeks**	mean **0.15 kg/m2 higher** (0.05 lower to 0.36 higher)	-	151 (4 RCTs)	⊕⊕⊕◯ Moderate[b]	The evidence is uncertain about the effect of mixed exercise on body Composition assessed by muscle mass index.
**Body mass composition: Fat mass assessed with: Fat mass percentage follow-up: range 8 weeks to 24 weeks**	mean **1.49 % lower** (3.21 lower to 0.23 higher)	-	189 (4 RCTs)	⊕⊕⊕⊕ High[c]	Mixed exercise results in a reduction in body mass composition: Fat mass percentage by -1.49% (95 CI -3.21, 0.23). Heterogeneity I^2= 31%
**Physical performance assessed with: Gait Speed follow-up: range 8 weeks to 24 weeks**	mean **0.08 m/s higher** (0.03 higher to 0.14 higher)	-	186 (4 RCTs)	⊕⊕⊕⊕ High	Mixed exercise results in large increase 0.07m/s (IC 95%: 0.00, 0.14) in physical performance assessed by Gait Speed. Heterogenity I^2= 41%.
**Strength assessed with: Handgrip strength follow-up: range 8 weeks to 24 weeks**	mean **2.21 kg higher** (0.02 higher to 4.41 higher)	-	221 (6 RCTs)	⊕⊕⊕◯ Moderate[c]	The evidence is very uncertain about the effect of mixed exercise on strength assessed by handgrip strength.
**GRADE Working Group grades of evidence<br>High certainty: we are very confident that the true effect lies close to that of the estimate of the effect.<br>Moderate certainty: we are moderately confident in the effect estimate: the true effect is likely to be close to the estimate of the effect, but there is a possibility that it is substantially different.<br>Low certainty: our confidence in the effect estimate is limited: the true effect may be substantially different from the estimate of the effect.<br>Very low certainty: we have very little confidence in the effect estimate: the true effect is likely to be substantially different from the estimate of effect.**					

a.Downgraded one level for risk of bias unclear
b.Downgraded one level because intervals include the possibility of a small or no effect
c.Downgraded one level for inconsistency due to I2= 66%
CI: confidence interval

© 2022. González-Rocha A et al. Published online at http://www.aginganddisease.org/EN/10.14336/AD.2022.0215
